# Investigation of the limits of high-definition muography for observation of Mt Sakurajima

**DOI:** 10.1098/rsta.2018.0135

**Published:** 2018-12-10

**Authors:** László Oláh, Hiroyuki K. M. Tanaka, Gergő Hamar, Dezső Varga

**Affiliations:** 1 Earthquake Research Institute, University of Tokyo, 1-1-1 Yayoi, Bunkyo, Tokyo 113-0032, Japan; 2 Wigner Research Centre for Physics of the Hungarian Academy of Sciences, 29-33 Konkoly-Thege Miklós Str., Budapest H-1121, Hungary

**Keywords:** muography, volcanology, muon, multiple scattering, gaseous detector, MWPC

## Abstract

A multi-wire proportional chamber-based muo- graphy observatory is under development for the monitoring of the internal structure of Mt Sakurajima in Kyushu, Japan. We investigated the limits of large-scale and high-definition muography. We adjusted the parameters of a modified Gaisser model and found that the spectral index of *γ* =  − 2.64 and normalization factor of *C* = 0.66 reproduce more accurately the measured fluxes than the original parameters at large thickness. A thickness and zenith angle-dependent correction is suggested to the measured muon flux due to the energy cut which is introduced to suppress the background particles. The multiple scattering of muons was simulated across the standard rock and sea-level atmosphere up to the distance of 5 km. We found that multiple scattering decreases from 10 mrad to 4 mrad across the rock due to the decrease in the steepness of muon spectra. The multiple scattering falls down to about 2 mrad after the object in the atmosphere due to the increase in observed arrival zenith angles. The 2 m^2^ sized multi-wire proportional chamber-based Muographic Observation System (MMOS) was operating between February and June 2018. Three tracking systems operated reliably with tracking efficiencies of above 95%. The muon flux has been measured correctly down to 10^−3^ m^−2^ sr^−1^ s^−1^. The average density map of Mt Sakurajima has been measured with angular resolution of 12 mrad × 12 mrad (spatial resolution of 34 m × 34 m from the distance of 2.8 km). The average density values were found between 1.4 and 2 g cm^−3^, except at the crater regions where lower densities were observed.

This article is part of the Theo Murphy meeting issue ‘Cosmic-ray muography’.

## 1. Introduction

Muography is a novel technique for the visualization of the internal structure of large objects [1]. The imaging process of muography is similar to radiography: tracking detectors are installed around an object and the absorption rate of penetrated cosmic-ray muons is measured. If the object is modelled, the average density length can be deduced by the comparison between measured and expected fluxes. Measurements from different directions allow tomographic imaging [2]. In addition, the newly developed airborne muography is not restricted by local topology and the imaging resolution of computational axial tomography can be achieved [3]. The applicability of cosmic-ray muography has been demonstrated to explore the internal structure of natural formations [2–11] and for inspection of human-made constructions [12–14].

**Figure 1.**
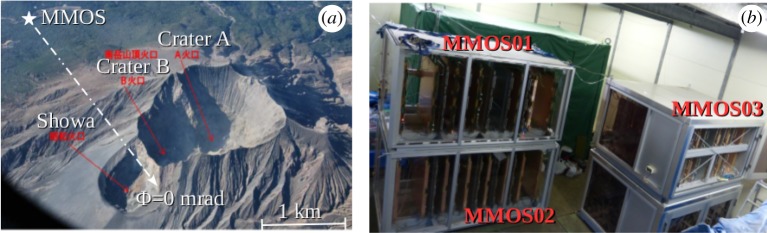
The Sakurajima muography campaign. (*a*) Photograph of Mt Sakurajima with the Craters A, B and Showa [15]. The location and orientation of MMOS are shown by a *star* and an *arrow*, respectively. (*b*) Photograph of the MMOS with three tracking systems in February 2018. (Online version in colour.)

The Hungary–Japan Joint Muography Observatory is focusing on the investigation and observation of the internal structure of Mt Sakurajima, which is located in Kyushu, Japan (see figure 1). Mt Sakurajima is one of the most active volcanoes with hundreds of explosive eruptions every year. The time-sequential imaging of density variations below the erupting craters could help to understand volcanic activity. Furthermore, the construction of a database with the variation of muon flux across the crater regions would contribute to a complex eruption prediction system which combines different geophysical techniques, such as gas emission [16], seismograph [17], video [18], GPS [19], satellite-based SAR [20] or LIDAR [21].

Mt Sakurajima is a difficult target for muography because of the kilometre-thick crater regions, which result in a muon flux below 10^−2^ m^−2^ sr^−1^ s^−1^. The craters A and B (Minami-dake) have been imaged with the angular resolution of 3 mrad with the first module of the MWPC-based Muographic Observations System (MMOS) [11,22] from the distance of 2.8 km in the southwest direction from the Showa crater in 2017. However, the large-scale extension of MMOS is necessary to provide time-sequential muographic images of Mt Sakurajima. Besides the limited flux of muons, the accuracy of large-scale muography is influenced by the following factors.

(i) Physical effects: The creation of muons is influenced by the variation of pressure and temperature in the atmosphere [23,24]. The flux of penetrated muons after the investigated object is comparable with the flux of background particles [25], which are also detected from the direction of the object and result in overmeasured flux as well as underestimated density. The background consists of low-energy muons [26,27], soft [28] and hadronic components of cosmic-rays. Besides the backround particles, the multiple scattering of muons across the investigated object causes a blurring effect on the muographic image.
(ii) Accuracy of muon flux modelling: The expected flux is determined by the integration of altitude corrected muon spectra from minimal energy which is necessary to penetrate the object or by Monte Carlo simulation of air showers across the atmosphere [23]. For the former approach, different muon spectra parametrizations [29–33] have been developed based on measurements performed in different energy ranges, see e.g. [34,35]. These discrepancies result in the differences of 10–30% between the different spectra models [23] and between the expected and measured fluxes [31,36,37]. The refining of muon spectra parametrization using world data could improve the accuracy of the calculated flux.
(iii) The detector effect: This depends on the applied technology (e.g. scintillator, nuclear emulsion, gaseous detector) as well as data acquisition (DAQ) and readout systems. Shielding plates are applied between the detector layers to deflect and absorb the background particles. The application of shielding is practically a momentum (energy) cut. The determination of an appropriate energy cut and the optimization of detector arrangement is suggested for each experiment.

This paper aims to improve the accuracy of muon flux calculation and measurement for large-scale muography, quantify the effect of multiple scattering of muons on the imaging resolution and give a report on the present status of the Hungary–Japan Joint Muography Observatory at the Sakurajima volcano. The paper is organized as follows. §2 focuses on the energy spectra of muons. §3 investigates the multiple scattering of muons. §4 focuses on the present status of the Sakurajima muography campaign. The results are summarized in §5.

## 2. Investigation of muon spectra for large-scale imaging

### (a) Adjustment of muon spectra parametrization

For large-scale muography, the high-energy region of the muon spectra has to be modelled accurately. These are described with the so-called modified Gaisser parametrization [29,30,33]:
2.1
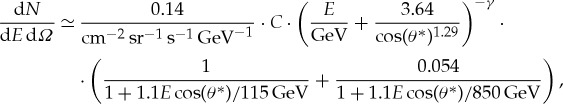
where d*N*/d*E* d*Ω* is the differential flux of muons, *C* = 1 is an adjustable constant factor, *γ* = − 2.7 is the spectral index inherited from the spectra of primary cosmic-rays. The 115 GeV and 850 GeV are critical energies for pions and Kaons, above these energies, the pions and Kaons interact with the atmosphere before they decay. The 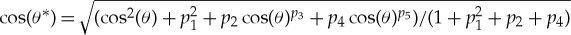 correction takes into account the curvature of the atmosphere and extends the zenith angle range of the parametrization [30,38] with the constant parameters of *p*_1_ = 0.102573, *p*_2_ = 0.068287, *p*_3_ = 0.958633, *p*_4_ = 0.0407253 and *p*_5_ = 0.817285. In addition, low-energy and high-energy muon spectra parametrizations have been introduced in [31,32], respectively.

Various groups calculated and simulated the near-vertical flux of muons based on parametrized spectra for underground experiments and reported 10–30% differences between their results and experimental data [31,36,37]. The original Gaisser parametrization has been refined and *C* = 0.805, *γ* =  − 2.643 parameters have been suggested to reproduce accurately the so-called Crouch curve [36]. The comparison of modelled fluxes to near-horizontal experimental data is suggested to improve the accuracy of large-scale muography.

**Figure 2.**
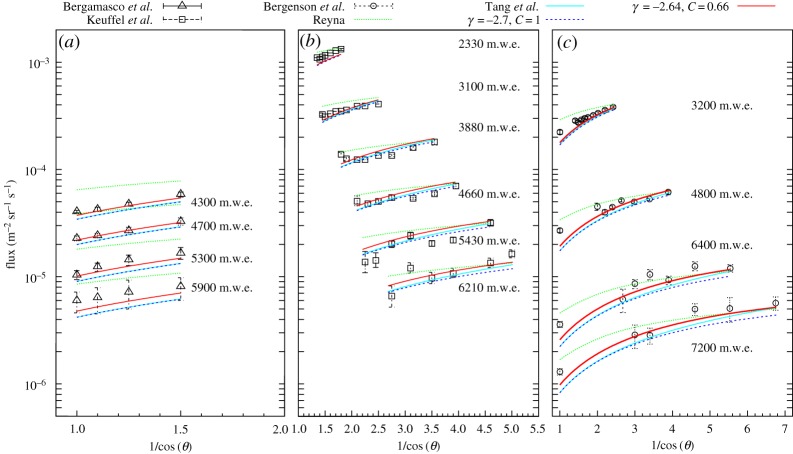
(*a*–*c*) The muon flux is plotted as the function of the zenith angle at different depths for experimental data (*black empty dots*), for the original (*blue dashed line*) as well as for optimal (*red solid line*) parametrization, respectively. Further models are drawn with *green* and *cyan* lines. The coloured lines on the calculated points are drawn to guide the eyes.

For this study, the muon spectra have been parametrized according to equation (2.1) and integrated from minimum energies based on CSDA range [39] for different thickness of standard rock at different zenith angles. The integrated flux values have been compared to fitted experimental data measured deep underground [40–42]. Figure 2 summarizes the experimental data (*black empty dots*) and the calculated flux values with the original (*blue dashed line*) and best fitting (*red solid line*) parametrizations. The parametrizations presented in [31,32] are also plotted with *dashed green line* and *solid cyan line*, respectively. We found that the modified Gaisser parametrization with *γ* = − 2.64 and *C* = 0.66 parameters produces a minimal difference between the expected fluxes and experimental data. The average values of the relative flux differences, (*F*_measured_ − *F*_expected_)/*F*_measured_, were found to be 10.4% and 1.6% for the original (*blue dashed line*) and for the best fitting (*red solid line*) parametrization, respectively.

### (b) An energy dependent correction to the flux penetrated muons

The suppression of low-energy background particles is required for large-scale muography. The energy spectra of background particles were found to be different at different measurement sites, e.g. they were below 1 GeV at Showa–Shinzan lava dome [25] and below 5 GeV at La Soufriére volcano [27]. These results suggest target specific investigation of background to determine the required energy cut and optimal detector arrangement. This section aims to investigate the reduction of the flux of penetrated muons due to the introduced energy cut independently from detector arrangement.

A simulation code based on GEANT4.10.01 [43] has been developed for this study. For each simulation, the object was implemented with different thicknesses between 250 and 2000 m standard rock equivalent (m.s.r.e.). The muons were injected near-horizontally (*θ*_0_≥70°) across the object and their energy spectra were parametrized according to Reyna and modified Gaisser models. A total of 40 000 muons were generated in each run. The minimum vertex energies of muons were set well below the minimum energies [39], which are required to penetrate the object. The application of lower energy minima was motivated by the energy loss of high-energy (>500 GeV) muons, which is dominated by stochastic processes (pair production, Bremsstrahlung, nuclear interaction) and some of the lower energy muons can also penetrate the object. The standard physics list with all of the relevant electromagnetic processes and muon nuclear processes were applied [44].

**Figure 3.**
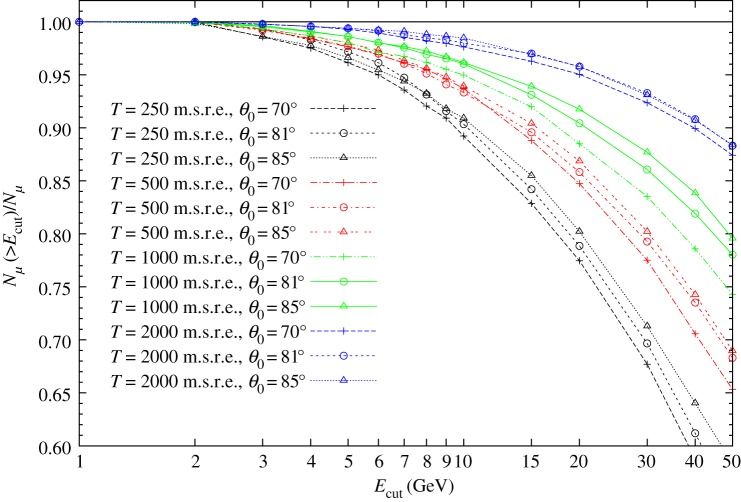
The ratio of the number of detected muons to the number of penetrated muons as the function of energy cut.

Figure 3 shows the ratio of the number of muons above the applied energy cut to the total number of penetrated muons, *N*_*μ*_( > *E*_cut_)/*N*_*μ*_, as the function of energy cut for various thicknesses and zenith angles. The application of an energy cut of a few GeV decreases the number of penetrated muons by only a few per cent, which is consistent with the results of [25,27]. Therefore, large-scale muography can be performed with a muon flux correction of a few per cent. The *N*_*μ*_( > *E*_cut_)/*N*_*μ*_ depends on thickness because of the decrease in the steepness of muon spectra at larger energies (relatively more energetic muons penetrate across thicker objects). A slight zenith angle dependence is also observed.

## 3. Investigation of muon scattering for high-definition muography

The spatial resolution of a muographic experiment, Δ*x*_imaging_, is given by the angular resolution of MOS, Δ*α*_MOS_ and the object-detector distance, *L*_air_: Δ*x*_imaging_ ≈ Δ*α*_MOS_ × *L*_air_. Therefore, the desired imaging resolution and the accessibility of the object (e.g. it is prohibited to approach Mt Sakurajima closer than 2.5 km) determine the expected angular resolution of MOS. Newly developed, versatile and robust detector technologies allow imaging resolution of below 10 mrad [11,14]. This section aims to investigate the multiple scattering of muons and quantify the upper limit of the angular resolution of muography.

The muons are deflected across the transversed object due to a series of Coulomb scatterings on the nuclei of the medium. The distribution of scattering angles after a small part of medium (0.001 < *L*/*X*_0_ < 100) follows a zero-centred Gaussian distribution [45] with a long tail and its root mean squared (RMS) value, *σ*_MS_, is approximated as follows:
3.1
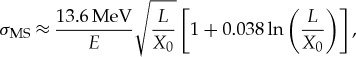
where *E* is the energy of muons and *L*/*X*_0_ is their path-length across the medium in radiation length units. The total scatterings of muons across the medium are given by the sum of small scatters along their path. The muons are losing continuously their energy across the medium, thus their scattering angles are increasing continuously across the medium.

**Figure 4.**
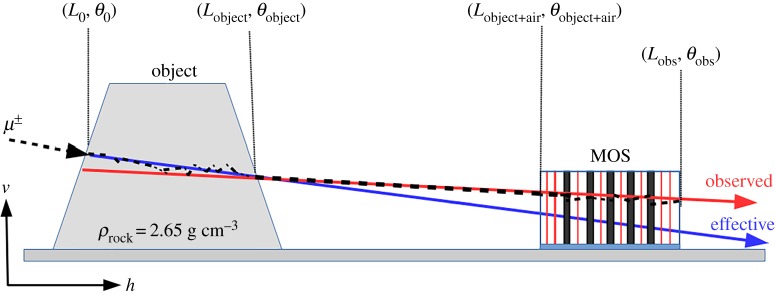
The schematic drawing of land-based muography. The multiple scattering of a muon across the object causes that its observed trajectory (*red arrow*) differs from the effective one (*blue arrow*) which carries information about the amount of material along the path of muon across the object. (Online version in colour.)

The multiple scattering cause the observed directions of penetrated muons (*red arrow* in figure 4) to differ from the effective paths (*blue arrow* in figure 4) along which they carry information about the amount of transversed material. The multiple scattering of muons in the air, *σ*_air_ and across the tracking system, *σ*_MOS_, have to be taken into account for long-range muography. The angular resolution of muography is suggested to be chosen above the total multiple scattering to avoid a smearing effect on the images:
3.2
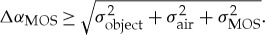
We note that the background particles also smear the muographic images. In the above criterion, we assume that the background particles are suppressed in the tracking system.

The sea-level atmosphere and a tracking system were also implemented in the above presented simulations (see in §2b) to study the multiple scattering of muons. The muons were tracked from the vertex to the end of the tracking system and their position vector, momentum direction vectors, energies were recorded in every 100 m across large-scale (≥500 m.s.r.e.) objects and in every 10 m across medium-scale ( ≤ 250 m.s.r.e.) objects, respectively. The geometrical arrangement of the simulations is presented in figure 4. An object made up from standard rock (i.e. mixture of MgCO_3_ and CaCO_3_ with density of 2.65 g cm^−3^) and a 2 m length MOS with eight MWPCs and five 2 cm thick lead plates were installed. The atmosphere was composed of N_2_ (78.1%), O_2_ (21%) and Ar (0.9%) and the pressure and temperature were set according to sea-level data (https://ccmc.gsfc.nasa.gov/modelweb/atmos/us_standard.html (last view 27/07/2018).). The average terrestrial magnetic field of *B* = 45 μT was also applied.

A track-by-track analysis has been performed on the simulated data to quantify the multiple scattering of muons. The analysis was initiated with the calculation of the projected scattering angles of muons. These were defined by the differences between the expected and observed arrival zenith angles. The projected scattering angles before the MOS (Δ*θ*_object+air_), across the atmosphere (Δ*θ*_air_) and across the MOS (Δ*θ*_MOS_) are described by the following equations:
3.3
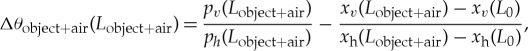

3.4
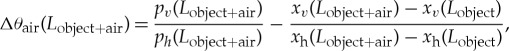

3.5
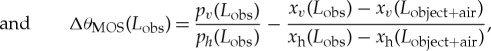
where *p*_h_ and *p*_v_ denote the horizontal and vertical components of the momentum direction vector; *x*_h_ and *x*_v_ denote the horizontal and vertical components of position coordinates (see in figure 4).

**Figure 5.**
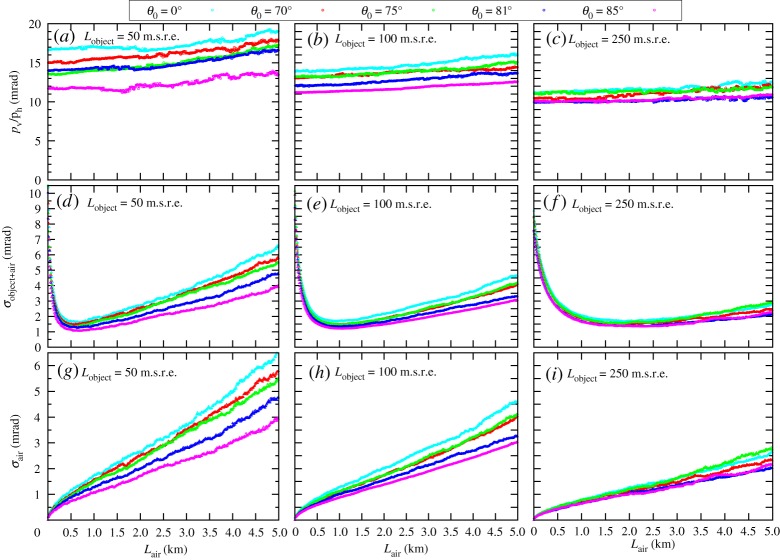
The multiple scattering of muons across medium-scale ( ≤ 250 m.s.r.e.) objects. The ratio of horizontal component to vertical component of momentum direction vector (*a*–*c*), the multiple scattering before the MOS (*d*–*f*) and across the air (*g*–*i*) are plotted as the function of object-MOS distance.

**Figure 6.**
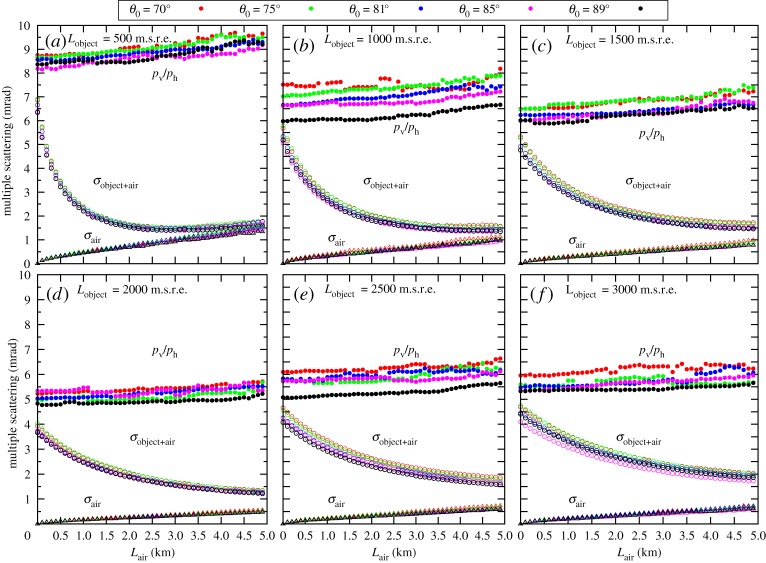
The multiple scattering of muons across large-scale objects. The ratio of horizontal component to vertical component of momentum direction vector (*p*_v_/*p*_h_), the multiple scattering before the MOS (*σ*_object+air_) and across the air (*σ*_air_) are plotted as the function of object-MOS distance for 500 m.s.r.e. (*a*), 1000 m.s.r.e. (*b*), 1500 m.s.r.e. (*c*), 2000 m.s.r.e. (*d*), 2500 m.s.r.e. (*e*) and 3000 m.s.r.e. (*f*), respectively.

Finally, the RMS values were calculated for the central 98% of the scattering angle distributions to determine the multiple scattering values for different locations before the tracking system. Figures 5 and 6 show the multiple scattering of muons after different objects up to the distance of 5 km. The results are summarized as follows.

(i) Both expected scattering angle of muons (*p*_v_/*p*_h_) and multiple scattering (*σ*_object+air_) decreases with the rock thickness from 15 mrad to 5 mrad and from 10 mrad to 4 mrad, respectively. The observed decrease in multiple scattering is caused by the decrease in the steepness of muon spectra. We note that tracking systems are placed in limited space in case of underground muography, thus multiple scattering can be approximated with *σ*_object+air_(*L*_air_ = 0). Based on this observation, the angular resolution of 10 mrad is suggested for portable devices applied for underground muography.
(ii) The multiple scattering (*σ*_object+air_) falls after an object in the atmosphere due to the increase of the observed arrival zenith angle (Δ*x*_v_/Δ*x*_h_). Consequently, it is optimal to install the MOS at a larger distance from the object to decrease the effect of multiple scattering. In the case of medium-sized objects, there is an optimal distance between 0.1 and 1 km where the effect of multiple scattering is minimal, and beyond this optimal distance the contribution of air (*σ*_air_) dominates.
(iii) As it is expected, *σ*_air_ is increasing with the distance from the object. This effect and its contribution to the total multiple scattering (*σ*_object+air_) decreases with the thickness of the object due to the decrease in the steepness of muon spectra.
(iv) The difference between the same type of quantities under different zenith angles is less than 10%, thus multiple scattering across large-scale objects has a slight dependence on the zenith angle of muons.
(v) The multiple scattering of detected muons across the MOS (*σ*_MOS_) is found to be ∼3.5 mrad, which is consistent with our previous work [11]. The *σ*_MOS_ depends on the positional resolution of tracking layers, the length of the tracking system and the amount of shielding plates, and thus it should be determined for each experiment.

**Figure 7.**
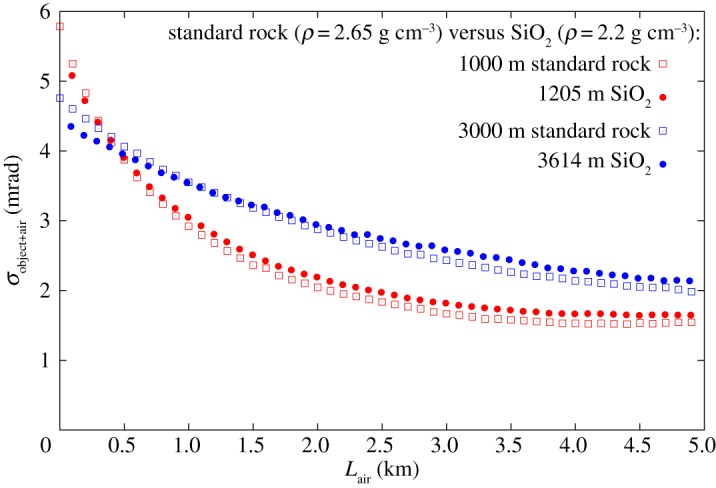
Comparison of the multiple scattering of 75° muons after standard rock and SiO_2_ with the same density length. (Online version in colour.)

The systematics of the simulations have also been investigated. The systematic errors caused by the Earth's magnetic field and physical properties of the atmosphere were found to be negligible. The chemical composition (*Z*, *A*) of rock was found to be a significant source of systematics errors. Figure 7 shows the multiple scattering of 75° muons after standard rock and SiO_2_ with the same density length of 1000 m.s.r.e. and 3000 m.s.r.e., respectively. The differences between the different compositions were found to be 5–10%. The observed differences are caused by the pair production and Bremsstrahlung energy loss processes which are scaled with *Z*^2^/*A*.

## 4. The MWPC-based Muography Observatory at Mt Sakurajima

An MWPC-based Muographic Observation System has been under development at the Sakurajima volcano since January 2017. The first high-definition images of Minami-dake are presented in [11]. The MMOS has been extended with two tracking systems to the sensitive surface of 2 m^2^ in February 2018. Each MMOS has at least five MWPCs and five 2 cm thick lead plates. There are 20 MWPCs in the three tracking systems. The length of each tracking system is 2 m. The tracking systems are housed in steel containers and isolated from the environment with Plexiglass walls. A non-flammable and non-toxic Ar-CO_2_ gas mixture (Ar 80: CO_2_ 20) is supplied parallel to the tracking systems with the flow of 2 l h^−1^. The electrical supply is provided by +110 V AC which is converted to +12 V DC. Each tracking system is protected by a +12 V (50 Ah) lead gel battery against power cuts. The total power consumption of MMOS is found below 30 watts (below 10 Watts per MMOS).

**Figure 8.**
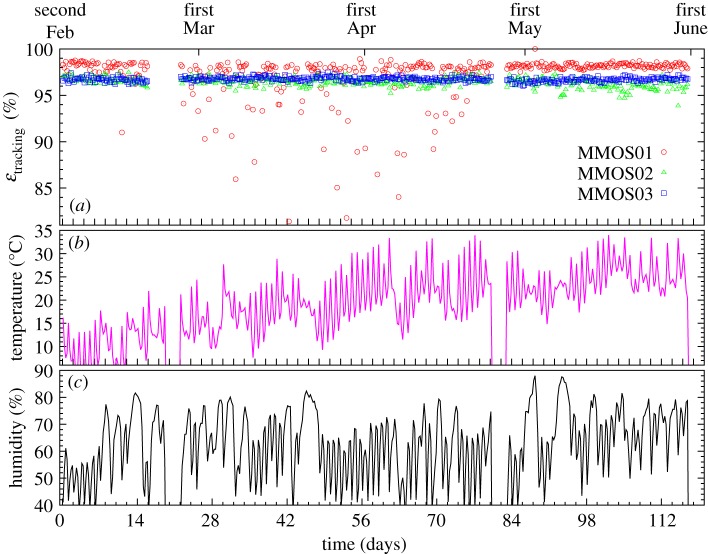
The variation of tracking efficiencies (*ε*_tracking_) of the three tracking systems (*a*), the temperature (*b*) and the humidity (*c*) during the data taking period between 2 February and 1 June. (Online version in colour.)

The tracking systems are operated independently from each other by microcomputer-based DAQ and control systems. The data acquisition process and the system are briefly presented in [11,22]. The MWPCs are supplied with a high-voltage of +1750 V to achieve a reasonable gas gain of above 10^3^. The trigger is provided by the triple coincidence of MWPCs. The front-end cards amplify the analogue signals and transfer the bits to the DAQ with shift registers. The data are compressed and transfered via a virtual private network to a remote server in every hour. Track reconstruction and track-by-track analysis are performed based on the analysis presented in [11]. The track rates, muon fluxes, are monitored on the remote server. Figure 8 shows the variations of tracking efficiencies (*ε*_tracking_) and environmental parameters during the period from 2 February to 1 June 2018. The two missing periods correspond to two technical stops between 19 and 24 February and between 24 and 26 of April. The detectors operated reliably with *ε*_tracking_ > 95% during the measurement period of 4 months, except MMOS01 which suffered from electrical noise due to the daily variation of humidity.

All tracking systems have to be oriented to the same direction within the imaging resolution before merging of their fluxes. An off-line correction method was developed for the orientation of tracking systems. First of all, the fluxes were calculated for each MMOS with the angular bin size of 6 mrad × 6 mrad. Thereafter, each flux was compared to the flux of the reference tracking system (MMOS03) and the flux residuals were calculated in different detector orientations. The residuals were found minimal when the tracking systems were oriented to the direction of MMOS03. An azimuthal rotation of 12 mrad and 30 mrad was applied to MMOS01 and MMOS02, respectively.

**Figure 9.**
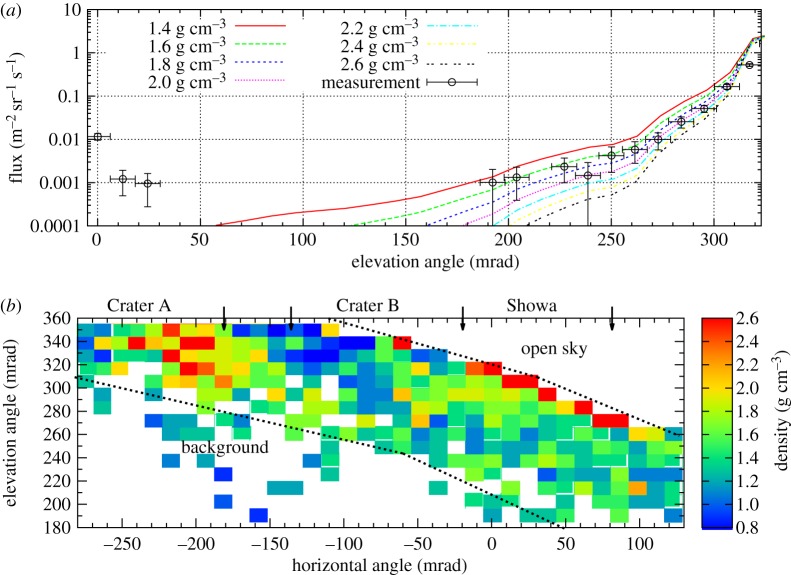
(*a*) The elevation angle dependence of muon flux at the horizontal angle of 0 mrad. (*b*) The density map of Mt Sakurajima with the angular bins of 12 mrad × 12 mrad (34 m × 34 m from the distance of 2.8 km).

The expected fluxes have been calculated with different densities using the modified Gaisser model (see in equation (2.1)) and the thickness map of Mt Sakurajima. Figure 9*a* shows the elevation angle dependence of the muon flux at the azimuth angle of 0 mrad with the angular bin size of 12 mrad. The flux of muons has been measured correctly down to 10^−3^ m^−2^ sr^−1^ *s*^−1^, where the contribution of background particles became dominant. The density map of Mt Sakurajima was also measured with the angular resolution of 12 mrad × 12 mrad (see in figure 9*b*). This angular resolution corresponds to the spatial resolution of 34 m × 34 m from the distance of 2.8 km. The average density values of Mt Sakurajima were observed to be between 1.4 g cm^−3^ and 2 g cm^−3^. The lower density regions (*blue patches*) show the structure of Minami-dake in the top region of Mt Sakurajima. The high-density region observed in Crater A is under investigation. The high-density regions in the downward-going ridge of the volcano are caused by the overestimation of muon flux by the high-energy spectra model. The relative errors of density values were found to be 10–20% between the *dashed black lines*. The Showa crater erupted only three times during the presented measurement period, thus these data can be used as a reference data set for time-sequential measurements of muon fluxes and average densities in the crater region.

## 5. Summary

An MWPC-based muography observatory is under development for the monitoring of the internal structure of Mt Sakurajima. This paper aimed to investigate the limits of large-scale and high-definition muography and present the status of the Sakurajima measurement campaign.

The energy spectra of muons have been investigated. We found that the modified Gaisser model reproduces more accurately the earlier experimental data at large rock thicknesses with *γ* = − 2.64 and *C* = 0.66 parameters than the original parametrization. The penetration of muons was simulated across standard rock to investigate the reduction of muon flux due to the energy cut applied to suppress the background particles. We found that the number of penetrated muons decreases a few per cent with the application of energy cut of few GeV. A thickness and angular-dependent correction of muon flux is recommended to experiments which apply energy cuts above few GeV. The multiple scattering of muons has been investigated to determine the upper limit of angular resolution of muography. We found that multiple scattering decreases from 10 mrad to 4 mrad with rock thickness due the decrease in the steepness of energy spectra and it falls after an object in the the atmosphere to about 2 mrad due to the increase of the observed arrival zenith angles.

The 2 m^2^ sized MMOS operated reliably with tracking efficiencies of above 95% between February and June 2018. The flux of penetrated muons was measured correctly down to 10^−3^ m^−2^ sr^−1^ s^−1^. The craters of Mt Sakurajima were visualized with spatial resolution of 34 m × 34 m from a distance of 2.8 km. The average density values of Mt Sakurajima were found between 1.4 g cm^−3^ and 2 g cm^−3^. The Showa crater erupted only three times during the measurement period, thus these data can be used as a reference dataset for time-sequential imaging of flux and density variations during active periods. The improvement and industrialization of MWPCs are ongoing to construct a large MMOS and allow time-sequential muography of Mt Sakurajima.

## Data accessibility

Data sets are available from the corresponding author on reasonable request.

## References

[1] Alvarez LW *et al.* 1970 Search for hidden chambers in the pyramids. Science 167, 832–839. (10.1126/science.167.3919.832) 17742609

[2] Tanaka HKM *et al.* 2010 Three-dimensional computational axial tomography scan of a volcano with cosmic ray muon radiography. J. Geophys. Res. 115, B12332 (10.1029/2010jb007677)

[3] Tanaka HKM 2016 Instant snapshot of the internal structure of Unzen lava dome, Japan with airborne muography. Sci. Rep. 6, 39741 (10.1038/srep39741) 28008978 PMC5180201

[4] Oláh L, Barnaföldi GG, Hamar G, Melegh HG, Surànyi G, Varga D 2012 CCC-based muon telescope for examination of natural caves. Geosci. Instrum. Method Data Syst. 1, 229–234. (10.5194/gi-1-229-2012)

[5] Miyamoto S *et al.* 2017 Muography of 1949 fault in La Palma, Canary Islands, Spain. Ann. Geophys. 60, 0110 (10.4401/ag-7385)

[6] Nishiyama R *et al.* 2017 First measurement of ice-bedrock interface of alpine glaciers by cosmic muon radiography. Geophys. Res. Lett. 44, 6244–6251. (10.1002/2017gl073599)

[7] Tanaka HKM *et al.* 2007 High resolution imaging in the inhomogeneous crust with cosmic-ray muon radiography: the density structure below the volcanic crater floor of Mt. Asama, Japan. Earth Planet. Sci. Lett. 263, 104–113. (10.1016/j.epsl.2007.09.001)

[8] Tanaka HKM, Uchida T, Tanaka M, Takeo M, Oikawa J, Ohminato T, Aoki Y, Koyama E, Tsuji H 2009 Detecting a mass change inside a volcano by cosmic-ray muon radiography (muography): first results from measurements at Asama, Japan. Geophys. Res. Lett. 36, L17302 (10.1029/2009gl039448)

[9] Carloganu C *et al.* 2013 Towards a muon radiography of the Puy de Dôme. Geosci. Instrum. Method. Data Syst. 2, 55–60. (10.5194/gi-2-55-2013)

[10] Tanaka HKM, Kusagaya T, Shinohara H 2014 Radiographic visualization of magma dynamics in an erupting volcano. Nat. Commun. 5, 3381 (10.1038/ncomms4381) 24614612 PMC3959196

[11] Oláh L, Tanaka HKM, Ohminato T, Varga D 2018 High-definition and low-noise muography of the Sakurajima volcano with gaseous tracking detectors. Sci. Rep. 8, 3207 (10.1038/s41598-018-21423-9) 29453335 PMC5816673

[12] Perry J *et al.* 2013 Imaging a nuclear reactor using cosmic ray muons. J. Appl. Phys. 113, 184909 (10.1063/1.4804660)

[13] Saracino G *et al.* 2017 Imaging of underground cavities with cosmic-ray muons from observations at Mt. Echia (Naples). Sci. Rep. 7, 1181 (10.1038/s41598-017-01277-3) 28446789 PMC5430851

[14] Morishima K *et al.* 2017 Discovery of a big void in Khufu's Pyramid by observation of cosmic-ray muons. Nature 552, 386–390. (10.1038/nature24647) 29160306

[15] Fukuoka District Meteorological Observatory, Regional Volcano Monitoring and Alarm Center, and Kagoshima Local Meteorological Observatory. 2017. *Volcanic activity of Sakurajima in Heisei era (2017)*. Tokyo, Japan: Japan Meteorological Agency. [in Japanese.]

[16] Kazahaya R, Shinohara H, Mori T, Iguchi M, Yokoo A 2016 Pre-eruptive inflation caused by gas accumulation: insight from detailed gas flux variation at Sakurajima volcano, Japan. Geophys. Res. Lett. 43, 11 219–11 225. (10.1002/2016gl070727)

[17] Kawakatsu H, Ohminato T, Ito H, Kuwahara Y, Kato T, Tsuruga K, Honda S, Yomogida K 1992 Broadband seismic observation at the Sakurajima Volcano, Japan. Geophys. Res. Lett. 19, 1959–1962. (10.1029/92gl01964)

[18] Cimarelli C, Alatorre-Ibargüengoitia MA, Aizawa K, Yokoo A, Díaz-Marina A, Iguchi M, Dingwell DB 2016 Multiparametric observation of volcanic lightning: Sakurajima Volcano, Japan. Geophys. Res. Lett. 43, 4221–4228. (10.1002/2015gl067445)

[19] Hickey J, Gottsmann J, Nakamichi H, Iguchi M 2016 Thermomechanical controls on magma supply and volcanic deformation: application to Aira caldera, Japan. Sci. Rep. 6, 32691 (10.1038/srep32691) 27619897 PMC5020646

[20] Morishita Y, Kobayashi T, Yarai H 2016 Three-dimensional deformation mapping of a dike intrusion event in Sakurajima in 2015 by exploiting the right- and left-looking ALOS-2 InSAR Japan. Geophys. Res. Lett. 43, 4197–4204. (10.1002/2016gl068293)

[21] Sakai T *et al.* 2014 Vertical distribution and optical properties of volcanic ash from Mt. Sakurajima detected with lidar and skyradiometer over saga. J. Remote Sens. Soc. Jpn. 34, 197–204. (10.11440/rssj.34.197)

[22] Varga D, Nyitrai G, Hamar G, Oláh L 2016 High efficiency gaseous tracking detector for cosmic muon radiography. Adv. High Energy Phys. 2016, 1962317 (10.1155/2016/1962317)

[23] Lesparre N, Gibert D, Marteau J, Déclais Y, Carbone D, Galichet E 2010 Geophysical muon imaging: feasibility and limits. Geophys. J. Int. 183, 1348–1361. (10.1111/j.1365-246x.2010.04790.x)

[24] Jourde K, Jourde K, Gibert D, Marteau J, de Bremond d'Ars J, Gardien S, Girerd C, Ianigro J-C 2016 Monitoring temporal opacity fluctuations of large structures with muon radiography: a calibration experiment using a water tower. Sci. Rep. 6, 23054 (10.1038/srep23054) 26971718 PMC4789792

[25] Nishiyama R, Taketa A, Miyamoto S, Kasahara K 2016 Monte Carlo simulation for background study of geophysical inspection with cosmic-ray muons. Geophys. J. Int. 206, 1039–1050. (10.1093/gji/ggw191)

[26] Jourde K, Gibert D, Marteau J, de Bremond d'Ars J, Gardien S, Girerd C, Ianigro J-C, Carbone D 2013 Experimental detection of upward going cosmic particles and consequences for correction of density radiography of volcanoes. Geophys. Res. Lett. 40, 6334–6339. (10.1002/2013gl058357)

[27] Gómez H, Gibert D, Goy C, Jourde K, Karyotakis Y, Katsanevas S, Marteau J, Rosas-Carbajal M, Tonazzo A 2017 Forward scattering effects on muon imaging. Jinst 12, P12018 (10.1088/1748-0221/12/12/p12018)

[28] Oláh L, Varga D 2017 Investigation of soft component in cosmic ray detection. Astropart. Phys. 93, 17–27. (10.1016/j.astropartphys.2017.06.002)

[29] Gaisser T 1990 Cosmic rays and particle physics. Cambridge, UK: Cambridge University Press.

[30] Chirkin D 2004 Fluxes of atmospheric leptons at 600 GeV–60 TeV. (http://arxiv.org/abs/hep-ph/0407078)

[31] Tang A, Horton-Smith G, Kudryavtsev VA, Tonazzo A 2006 Muon simulations for Super-Kamiokande, KamLAND, and CHOOZ. Phys. Rev. D 74, 053007 (10.1103/physrevd.74.053007)

[32] Reyna D 2006 A simple parameterization of the cosmic-ray muon momentum spectra at the surface as a function of zenith angle. (http://arxiv.org/abs/hep-ph/0604145)

[33] Guan MY, Chu M-C, Cao J, Luk K-B, Yang C 2015 A parametrization of the cosmic-ray muon flux at sea-level. (http://arxiv.org/abs/1509.06176)

[34] Jokisch H, Carstensen K, Dau WD, Meyer HJ, Allkofer OC 1979 Cosmic-ray muon spectrum up to 1 TeV at 75° zenith angle. Phys. Rev. D 19, 1368 (10.1103/PhysRevD.19.1368)

[35] Allkofer OC, Bella G, Dau WD, Jokisch H, Klemke G, Oren Y, Uhr R 1985 Cosmic ray muon spectra sea-level up to 10 TeV. Nucl. Phys. B 259, 1–18. (10.1016/0550-3213(85)90294-9) 9955872

[36] Reichenbacher J, De Jong J 2008 Calculation of the underground muon intensity crouch curve from a parametrization of the flux at surface. In *Proc. 30th ICRC*, vol. 5.

[37] Blyth SC *et al.* 2016 Measurement of cosmic-ray muons and muon-induced neutrons in the Aberdeen Tunnel Underground Laboratory. Phys. Rev. D 93, 072005 (10.1103/PhysRevD.93.072005)

[38] Volkova LV 1969 Lebedev Physical Institute Report No. 72. Moscow, Russia: Lebedev Physical Institute.

[39] Groom DE, Mokhov NV, Striganov SI 2002 Muon stopping power and range tables 10 MeV–100 TeV. At. Data Nucl. Data Tables 76, 183–356. (10.1006/adnd.2001.0861)

[40] Keuffel JW, Osborne JL, Bolingbroke GL, Mason GW, Larson MO, Lowe GH, Parker JH, Stenerson RO, Bergeson HE 1969 Zenith-angle distribution of ultra-high energy muons. In *Proc. 11th Int. Conf. on Cosmic Rays*, vol. 29.

[41] Bergenson HE, Bolingbroke GL, Carlson G, Groom DE, Keuffel JW, Morrison JL, Osborne JL 1971 Zenith-angle dependence of cosmic-ray muons. Phys. Rev. Lett. 27, 160–163. (10.1103/physrevlett.27.160)

[42] Bergamasco L, Castellina A, D'Ettorre Piazzoli B, Mannocchi G, Picchi P, Vernetto S, Bilokon H 1983 Muon sea-level intensity and primary cosmic-ray nucleon spectrum in the (1–100) TeV energy range from the Mt. Blanc underground experiment. Il Nou. Cim. C 6, 569–594. (10.1007/bf02507335)

[43] Agostinelli S *et al.* 2003 Geant4—a simulation toolkit. Nucl. Instr. Meth. A 506, 250–303. (10.1016/S0168-9002(03)01368-8)

[44] Bogdanov AG, Burkhardt H, Ivanchenko VN, Kelner SR, Kokoulin RP, Maire M, Rybin AM, Urban L 2004 Geant4 simulation of high energy muon interactions. In *IEEE Symp. Conf. Record Nuclear Science*, vol. 2004.

[45] Bethe HA 1953 Moliere's theory of multiple scattering. Phys. Rev. 89, 1256–1266. (10.1103/physrev.89.1256)

